# MZe786 Rescues Cardiac Mitochondrial Activity in High sFlt-1 and Low HO-1 Environment

**DOI:** 10.3390/antiox9070598

**Published:** 2020-07-09

**Authors:** Lissette Carolina Sanchez-Aranguren, Homira Rezai, Shakil Ahmad, Faisal A. Alzahrani, Anna Sparatore, Keqing Wang, Asif Ahmed

**Affiliations:** 1 Aston Medical Research Institute, Aston Medical School, Birmingham B4 7ET, UK; lcsanchez_7@hotmail.com (L.C.S.-A.); May@mirzyme.com (H.R.); s.ahmad@aston.ac.uk (S.A.); Keqing@gmail.com (K.W.); 2 Mirzyme Therapeutics, Innovation Birmingham Campus, Faraday Wharf, Holt Street, Birmingham B7 4BB, UK; 3 King Fahad Center for Medical Research, King Abdulaziz University, Jeddah 21589, Saudi Arabia; faahalzahrani@kau.edu.sa; 4 Department of Pharmaceutical Science, University of Milan, 20122 Milan, Italy; anna.sparatore@unimi.it

**Keywords:** heme oxygenase-1, sFlt-1, preeclampsia, mitochondria, antioxidants, hydrogen sulfide

## Abstract

Hypertensive disorder in pregnancy is a major cause of maternal and perinatal mortality worldwide. Women who have had preeclampsia are at three to four times higher risk in later life of developing high blood pressure and heart disease. Soluble Flt-1 (sFlt-1) is elevated in preeclampsia and may remain high postpartum in women with a history of preeclampsia. Heme oxygenase-1 (Hmox1/HO-1) exerts protective effects against oxidative stimuli and is compromised in the placenta of pregnant women with preeclampsia. We hypothesized that sFlt-1 inhibits cardiac mitochondrial activity in HO-1 deficient mice. HO-1 haplo-insufficient mice (Hmox1^+/−^) were injected with adenovirus encoding sFlt-1 (Ad-sFlt-1) or control virus (Ad-CMV). Subsequently, they were treated daily with either placebo or MZe786 for six days, when the heart tissue was harvested to assess cardiac mitochondrial activity. Here, we show that the loss of HO-1 disturbed cardiac mitochondrial respiration and reduced mitochondrial biogenesis. The overexpression of sFlt-1 resulted in the inhibition of the cardiac mitochondrial activity in Hmox1^+/−^ mice. The present study demonstrates that the hydrogen sulfide (H_2_S) releasing molecule, MZe786, rescues mitochondrial activity by stimulating cardiac mitochondrial biogenesis and antioxidant defense in Hmox1^−/−^ mice and in Hmox1^+/−^ mice exposed to a high sFlt-1 environment.

## 1. Introduction

Preeclampsia is a pregnancy-related complication that affects more than 10 million women a year and is the leading cause of maternal and perinatal morbidity and mortality worldwide [1,2,3]. It is also recognized as an independent risk factor for heart diseases. Heart disease and preeclampsia are a major economic burden on public health systems [4,5,6,7,8]. Women who have had preeclampsia are three to four times more at risk of developing high blood pressure and are twice as likely to develop heart disease, heart failure, and stroke later in life [9,10,11]. The risk of cardiovascular complications further increases in women with a history of preeclampsia and preterm delivery, low birthweight babies or those who have suffered from severe preeclampsia [10,12,13]. 

In the last decade, the hypothesis that preeclampsia arises due to “increase in the level of endogenous soluble Flt-1 (sFlt-1) that may antagonize the beneficial effects of vascular endothelial growth factor (VEGF)” [14] has been validated [15,16,17]. Several studies have consistently shown that the level of sFlt-1 is strongly associated with the clinical signs of preeclampsia and disease severity [15,18,19,20]. In preeclampsia, heme oxygenase-1 (Hmox1/HO-1) plays a protective role against the disease and is a negative regulator of sFlt-1 [21]. The adenoviral overexpression of HO-1 reduced sFlt-1 release from endothelial cells, while siRNA-mediated Hmox1 knockdown increases sFlt-1 release [22]. After birth, the level of circulating sFlt-1 decreases following the delivery of the placenta [23,24]. Interestingly, a study by Akhter et al., 2016 demonstrated that, at one year postpartum, the level of sFlt-1 was still higher in women who had preeclampsia than in the control group [25]. HO-1 plays a crucial role in cardiovascular homeostasis and has a cardiovascular protective function. The absence of HO-1 leads to accelerated atherosclerotic lesion formation [26] and exacerbated myocardial reperfusion injury [27]. In contrast, the cardiac-specific overexpression of HO-1 in mice protects against ischemia [28] and doxorubicin-induced cardiomyopathy [29]. 

Hydrogen sulfide (H_2_S) belongs to a family of labile biological mediators called gasotransmitters. It is synthesized by numerous mammalian tissues by three enzymes—cystathionine γ-lyase (CSE), cystathionine β-synthetase (CBS), and 3-mercaptopyruvate sulfurtransferase (3-MST) [30]. Many researchers have recently looked at the potential options for the therapeutic exploitation of hydrogen sulfide for its anti-inflammatory and cytoprotective properties through the preservation of mitochondrial function and the regulation of calcium homeostasis [31,32]. H_2_S inhibited oxidative stress through cysteine S-thiol to scavenge free radicals in the atherosclerosis mouse model [33] and activated the Nrf2 pathway in the Angiotensin-II-induced hypertension mouse model [34]. H_2_S has also been shown to promote vasodilatation [35], exhibit cytoprotective anti-inflammatory properties [36], protect against reperfusion injury-induced cellular damage [37], and stimulate angiogenesis [38]. Interplay between H_2_S and other gasotransmitters, such as HO-1 by-product, carbon monoxide (CO), was suggested to modulate vascular function. H_2_S may have a role in the control of CO bioavailability under specific pathophysiological conditions [39]. Human mesangial cells and human podocytes treated with H_2_S donor increased HO-1 and its by-product CO [40]. Another study by Zhang and colleagues investigated the role of H_2_S in volume overload-induced Chronic Heart Failure (CHF) in rats and demonstrated that H_2_S may play a protective role in volume overload-induced CHF by upregulating HO-1 [41].

MZe786 is a novel synthesis H_2_S-releasing molecule, which protects the gastric mucosa from its anti-cyclooxygenase activity [42]. H_2_S release helps to rebalance the redox system as a result of increased H_2_S/gluthathione (GSH) formation, increased HO-1 promoter activity and reduced 8-isoprostane. MZe786 has also been shown to improve endothelial function in animal models of ischemia-reperfusion injury, possibly by modulating levels of glutathione and homosysteine [43,44]. 

We have previously demonstrated using serum obtained from women with preeclampsia that increased levels of sFlt-1 leads to metabolic perturbations accountable for mitochondrial dysfunction in cultured endothelial cells. Furthermore, sFlt-1 exacerbated mitochondrial reactive oxygen species formation and mitochondrial membrane potential dissipation [45]. As the increased circulatory level of sFlt-1 is associated with cardiovascular complications, such as chronic heart failure [46,47], we sought to investigate whether HO-1 and sFlt-1 play a role in the regulation of cardiac mitochondrial activity using a well-established model of high sFlt-1 environment by the tail-vein injection of adenovirus sFlt-1 in Hmox1^+/−^ mice. We further tested whether a hydrogen sulfide-releasing molecule, MZe786, could rescue sFlt-1-induced mitochondrial dysfunction in the heart of Hmox1^−/−^ mice. 

## 2. Materials and Methods

### 2.1. Adenovirus Preparation

Recombinant adenovirus-encoding mouse sFlt-1 (Ad-sFlt-1) under the control of the CMV promoter was a gift from Professor Richard Mulligan (Harvard School of Medicine, Boston, MA, USA) and adenovirus containing the CMV promoter without an insert (Ad-CMV) was purchased from Vector Biolabs, Pennsylvania, USA and stored at −80 °C. 

### 2.2. Animal Studies

All animal experiments were carried out using procedures approved by the Aston University Ethical Review Committee in compliance with the UK Home Office License Number 3003453 in accordance with the “Guidance on the operation of Animals” under the United Kingdom Animals (Scientific Procedures) Act 1986.

### 2.3. Drug Preparation

MZe786 (2-acetyloxybenzoic acid 4-(3-thioxo-3H-1,2-dithiol-5-yl) phenyl ester; chemical structure of MZe786, as shown Figure S1, is a novel hydrogen sulfide-releasing drug comprising a H_2_S-releasing dithio-thione moiety, synthesized by Professor Anna Sparatore. The drug was prepared in a drug carrier (0.5% carboxymethyl cellulose in PBS) fresh every day.

### 2.4. Animal Experimental Protocol

Heterozygous Hmox1 (Hmox1^+/−^) mice were originally obtained from Professor Anupam Agarwal (University of Alabama, Birmingham, AL, USA). Female wildtype (Hmox1^+/+^), Hmox1^+/−^, or knockout (Hmox1^−/−^) mice between the ages of twelve to twenty weeks were used. Mice lacking HO-1 (Hmox1^−/−^ animals) received the drug carrier or 50 mg/kg of MZe786 treatment via gavage for six days before the heart was harvested for analysis. Animals were injected with 0.5 × 10^9^ PFU/mL Ad-sFlt-1 or empty vector (Ad-CMV) via the tail vein. Dose titration studies were used to determine the non-toxic dose of Ad-sFlt-1 and 0.5 × 10^9^ PFU increased sFlt-1 expression without inducing any toxic effect in mice. Mice were euthanized via cervical dislocation and the heart was harvested.

### 2.5. Isolation of Mitochondria

Mitochondria were isolated from mouse hearts by homogenization and density gradient centrifugation using a method adapted from Sakamuri et al. [48]. Mice were sacrificed and hearts isolated and collected in 5 mL mitochondrial isolation media (MIM) (70 mM sucrose, 210 mM mannitol, 5 mM HEPES, 1mM EGTA, pH 7.4) supplemented with 0.5% fatty acid-free BSA and kept at 4 °C. The following steps were conducted at 4 °C or on ice. Immediately after tissue harvesting, hearts were rinsed with MIM several times to remove the blood and fat tissue surrounding the organ, which was carefully removed using a scalpel and tweezers. The tissue was chopped and transferred to a Precellys tube containing silica beads. Following this, the tissue was homogenized in MIM using a VelociRuptor V2 Microtube Homogeniser (Scientific Laboratories Supplies, Nottingham, UK) for 10 s. The homogenate was transferred to a 15 mL polypropylene tube and centrifuged at 500× *g* for 5 min at 4 °C. Tissue supernatant was transferred to an OptiSeal Polypropylene Tube (Beckman Coulter, UK) and centrifuged at 10,000× *g* for 6 min at 4 °C. The pellet was re-suspended in 1.2 mL 15% Percoll in BSA-free MIM. In a separate tube, a gradient of Percoll was prepared by adding 1.2 mL 24% Percoll followed by 1.5 mL 40% Percoll (prepared in BSA-free MIM) added to the bottom of the tube. Next, the pellet was slowly layered on top of the 24% Percoll. A visible layer (mitochondria) was observed between the 24 and 40% Percoll layers after centrifuging the tube at 30,000× *g* for 8 min at 4 °C. The mitochondrial layer was collected using a 5 mL syringe needle and transferred to a new tube containing 2 mL BSA-free MIM and centrifuged at 16,000× *g* for 10 min at 4 °C. The pellet was transferred to a separate tube containing 5 mL BSA-free MIM and centrifuged at 7000× *g* for 10 min at 4 °C, after which the final pellet was collected and re-suspended in 50 μL BSA-free MIM. The total protein content was determined using the Bradford Assay Reagent (Bio-Rad, Watford, UK).

### 2.6. Mitochondrial Respiration Assays

Mitochondrial respiration was assessed using an XF24 Extracellular Flux Analyzer (Seahorse Bioscience, Billerica, MA, USA) using a method adapted from Boutagy et al. [49]. In preparation, 24 h prior to the respirometry assays, special XF24 sensor cartridges were hydrated overnight at 37 °C using Seahorse XF calibrant solution (800 μL per well) (Seahorse Bioscience, Agilent, Cheshire, UK). The day of the assay, cardiac mitochondria was isolated and plated in V7 Seahorse XF24 tissue culture microplates at a density of 10 μg per well in 50 μL mitochondrial assay solution (MAM) (70 mM sucrose, 210 mM mannitol, 2 mM HEPES, 1 mM EGTA, 5 mM MgCl_2_, 10 mM KH_2_PO_4_, 0.2% (w/v) fatty acid-free BSA, pH 7.4).

To effectively determine the mitochondrial activity in response to complex I and II substrates, MAM was supplemented with either: (a) 10 mM pyruvate/2 mM malate; (b) 10 mM succinate/2 μM rotenone; (c) 40 μM palmitoyl-L-carnitine/2 mM malate. MAM containing only substrate was loaded to blank wells A1, C3, B4, and D6. After plating the mitochondria, the microplate was centrifuged at 2000× *g* for 10 min at 4 °C to allow mitochondria to attach to the plate bottom. Then, 450 μL of pre-warmed MAM-containing substrate was added to the respective wells. Finally, the microplates were incubated for 10 min at 37 °C in a non-CO_2_ incubator before the measurement of mitochondrial respiration was started. Once in the XF24 Analyzer, oxygen consumption rates were calculated by plotting oxygen concentration (pmol O_2_) vs. time (min). Mitochondrial oxygen consumption was measured after the sequential injections of ADP 1 mM, 2 μM oligomycin (ATP synthase inhibitor), FCCP 4 μM, and antimycin A 4 μM. Oxygen consumption rates measured after ADP injections are representative of the maximal coupled respiration, or state 3. To determine the oxygen consumption rate following the ATP/ADP ratio approaching equilibrium, we measured the rates of oxygen consumption after oligomycin injection (state 4o). For these parameters, states 3 and 4o were used to quantify the behavior of mitochondria by calculating the RCR, calculated by dividing the respiration determined in state 3 by that in state 4o [50].

### 2.7. Quantitative Real-Time PCR

RNA was extracted from heart tissue using the RNeasy mini-kit (Qiagen, Hilden, Germany) and quantified using the Nanodrop ND 1000 spectrophotometer (NanoDrop Technologies Inc., Wilmington, Delaware, USA). RNA was converted to cDNA using the EvoScript Universal cDNA Master (Roche Life Sciences, Basel, Switzerland) as indicated by the manufacturers’ guidelines. The relative expressions of mouse genes—PGC1-α, thioredoxin (Txn1), glutaredoxin (Glrx), hexokinase 2 (HK2), and ND1—were quantified by real-time PCR on a Lightcycler 480 (Roche Life Sciences, Basel, Switzerland) using the LightCycler^®^ 480 SYBR^®^ Green I Master (Roche, Life Sciences, Basel, Switzerland) and its specific primers. RT-PCR was performed using the following run conditions: Pre-incubation (1 cycle); amplification (45 cycles); melting curve (1 cycle); cooling (1 cycle). Relative gene expression was calculated using the 2^−ΔΔCT^ method. A comparison of ND1 DNA expression, relative to HK2 DNA expression, provided a measure of the mtDNA copy number to nDNA copy number ratio (mtDNA/nDNA) [51].

### 2.8. Statistical Analysis

Data were plotted as means ± SEM. Statistical analysis was performed using Kruskal–Wallis with Dunn’s post hoc test, using GraphPad Prism software (GraphPad Software, San Diego, CA, USA). Values of *p* < 0.05, *p* < 0.01, and *p* < 0.001 were considered statistically significant.

## 3. Results

### 3.1. Loss of HO-1 Disturb the Cardiac Mitochondrial Respiration

HO-1 has been proposed to exert cytoprotective effects over oxidative stress-mediated hypertrophy, fibrosis, and metabolic dysregulation in heart tissue [27]. From a metabolic perspective, the heart is the most active organ in the body. To sustain its metabolic demands, cardiac tissue relies mainly on mitochondrial oxidative phosphorylation (OXPHOS) to produce ATP [52]. To study whether HO-1 has a role in the regulation of cardiac mitochondrial activity, we evaluated the mitochondrial bioenergetics function in isolated cardiac mitochondria from Hmox1^−/−^ mice. Using a XF24 Seahorse Analyzer, we evaluated the capacity of mitochondria for substrate oxidation by calculating the mitochondrial respiratory control ratio (RCR). The RCR is defined as the respiration in state 3 divided by that in state 4o [50]. 

**Figure 1:**
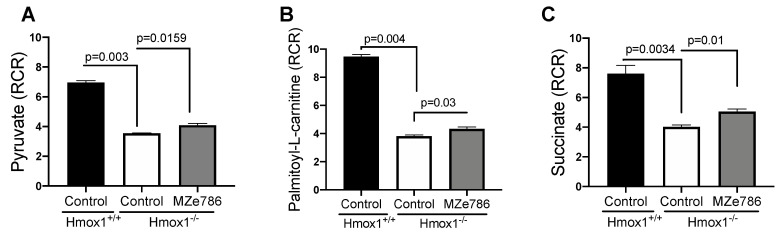
Loss of HO-1 disturb the cardiac mitochondrial respiration. Mitochondrial oxygen consumption was measured in isolated cardiac mitochondria from Hmox1^+/+^ and Hmox1^−/−^ mice exposed to MZe786 via gavage. The respiratory control ratio (RCR) (state 3/state 4o) was calculated using complex I-driven substrates: (**A**) pyruvate and (**B**) palmitoyl-L-carnitine. (**C**) RCR was calculated in complex II-stimulated cardiac mitochondria using succinate as substrate. Values are expressed as means ± SEM. *N* = 4.

First, we measured the ability of cardiac mitochondria for the oxidation of pyruvate and palmitoyl-L-carnitine at the mitochondrial complex I in the presence of malate (complex II inhibitor) by calculating the RCR. Oxygen consumption rates representative of state 3 and state 4o respiration were used to calculate the RCR, as shown in Figure S2. In response to pyruvate, cardiac mitochondria isolated from the Hmox1^−/−^ mice displayed a significant reduction in the calculated RCR when compared to Hmox1^+/+^ (*p* = 0.003). The administration of MZe786 to Hmox1^−/−^ mice showed a significant restoration in this parameter in comparison to Hmox1^−/−^ cardiac mitochondria (*p* = 0.0159), as shown in Figure 1A. Next, we evaluated the ability of cardiac mitochondria from Hmox1^−/−^ mice for the oxidation of palmitoyl-L-carnitine. Our results show a significant compromise in fatty acid oxidation in the absence of HO-1, as shown in Figure 1B, when compared to Hmox1^+/+^ (*p* = 0.004). Similar to pyruvate-dependent complex I activity, MZe786 was able to improve the oxidation of palmitoyl-L-carnitine in comparison to Hmox1^−/−^ mice (*p* = 0.03). Correspondingly, we explored the ability of cardiac mitochondria for the oxidation of succinate (complex II substrate) in the presence of rotenone (complex I inhibitor). State 3 and state 4o respiration was used to calculate the RCR, as shown in Figure S2. In contrast with wild type isolated cardiac mitochondria, we evidenced that Hmox1^−/−^ mitochondria are less able to respire using succinate as a substrate (*p* = 0.0034). Interestingly, MZe786 protected complex II-driven OXPHOS improved the calculated RCR when compared to Hmox1^−/−^ hearts (*p* = 0.01), as shown in Figure 1C. These results propose that the loss of HO-1 impairs complex I and II substrate oxidation, which resulted in the dysregulation of OXPHOS in the heart. Alongside this, the administration of MZe786 showed to improve the complex I and II electron transfer efficiency in the absence of HO-1.

### 3.2. MZe786 Improves Cardiac Mitochondrial Biogenesis Signal and Stimulates Antioxidant Gene Transcription in Hmox1 Knockout Mice

Once we established that the loss of HO-1 leads to signs of mitochondrial dysfunction in the heart, we sought to evaluate the role of MZe786 in the restoration of the cardiac mitochondrial function. As previously reported, peroxisome proliferator-activated receptor gamma coactivator 1-alpha (PGC1-α) plays a key role in cardiac metabolism by enhancing the mitochondrial biogenesis signal [53,54] and promoting the antioxidant defense system [55] in the heart. Therefore, we evaluated the gene expression of PGC1-α and the content of mitochondria expressed as the ratio of mitochondrial DNA by nuclear DNA (mtDNA/nDNA) in our model.

**Figure 2:**
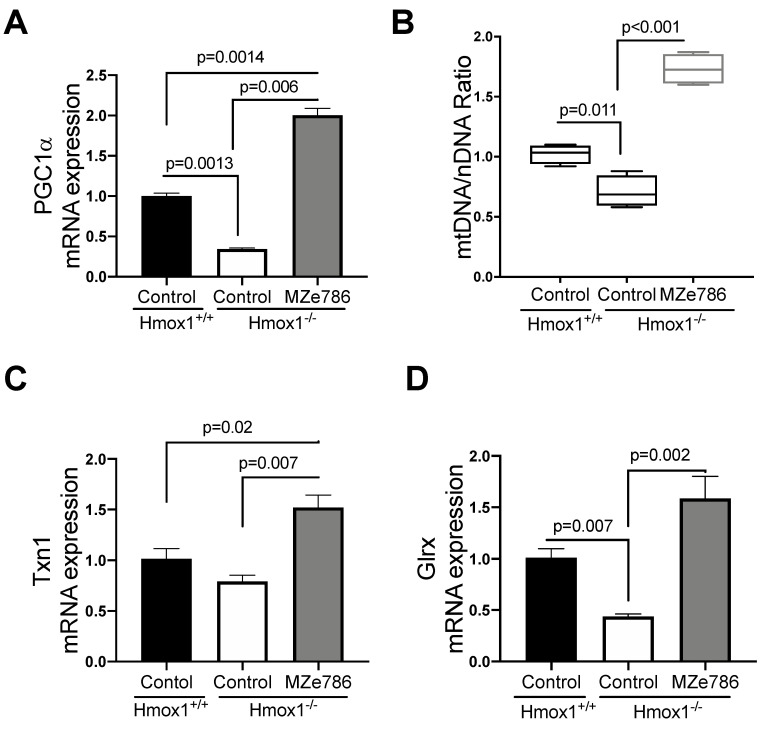
MZe786 stimulates antioxidant genes transcription in Hmox1 knockout mice. (**A**) Relative PGC1α mRNA expression, (**B**) content of mitochondria expressed as the ratio of mitochondrial DNA by nuclear DNA (mtDNA/nDNA). (**C**) Relative mRNA expression of antioxidant gene thioredoxin (Txn1) and (**D**) glutaredoxin (Glrx) measured by qPCR in heart tissue from Hmox1^+/+^ and Hmox1^−/−^ mice exposed to MZe786 via gavage. A, C and D values are expressed as means ± SEM. Values in B are expressed as median and whiskers represent maximum and minimum values. *N* = 4.

The loss of HO-1 significantly reduced the gene expression of PGC1-α in cardiac tissue in comparison to their wild type counterparts (*p* = 0.0013). MZe786 significantly increased PGC1-α transcription when compared to wild type (*p* = 0.0014) and Hmox1^−/−^ (*p* = 0.0006) hearts, as shown in Figure 2A. As expected, we evidenced a significant reduction in the mtDNA/nDNA ratio in Hmox1 knockout cardiac tissue in contrast to the wild type (*p* = 0.011), while MZe786 significantly enhanced the mtDNA/nDNA in Hmox1^−/−^ when compared to both Hmox1^+/+^ and Hmox1^−/−^ hearts (*p* < 0.0001), as shown in Figure 2B.

We next focused on the ability of MZe786 to stimulate antioxidant gene expression in the absence of HO-1. Our results showed a slight reduction in thioredoxin (Txn1) gene expression in Hmox1^−/−^ in comparison to Hmox1^+/+^ hearts (*p* = 0.12). The administration of MZe786 significantly stimulated Txn1 gene expression when compared to Hmox1^+/+^ (*p* = 0.02) and Hmox1^−/−^ (*p* = 0.007) cardiac tissue, as shown in Figure 2C. Consistently, the gene expression of glutaredoxin (Glrx) was compromised in Hmox1^−/−^ hearts (*p* = 0.007), while MZe786 was able to restore its expression (*p* = 0.002). There was no significant difference between cardiac Glxr gene expression the in wild type compared to MZe786-treated Hmox1^−/−^ mice (*p* = 0.14), as shown in Figure 2D. This suggests that the loss of HO-1 dysregulates the expression of antioxidant genes in cardiac tissue. Interestingly, MZe786 is able to promote the expression of both Txn1 and Glrx and proposes a role of MZe786 as an inducer of cardiac antioxidant defense in the absence of HO-1.

### 3.3. MZe786 Rescues the sFlt-1-Induced Inhibition of the Cardiac Mitochondrial Activity in Hmox1^+/−^ Mice

Haploinsufficiency of Hmox1 (Hmox1^+/−^, single Hmox1 allele) has been associated with the dysregulation of angiogenic balance, leading to signs of fetal growth restriction and preeclampsia [56]. Later in life, women who experienced preeclampsia have increased risk of developing heart disease in comparison to those experiencing normal pregnancies [57,58]. Therefore, we sought to explore whether the partial deficiency of HO-1 in a high sFlt-1 environment, mimicking preeclampsia-like settings, would disturb the mitochondrial function in cardiac tissue. We systemically injected adenovirus encoding sFlt-1 to female Hmox1^+/−^ mice and evaluated the respiratory efficiency in mitochondria isolated from heart tissue using an XF24 Seahorse Analyzer. 

**Figure 3:**
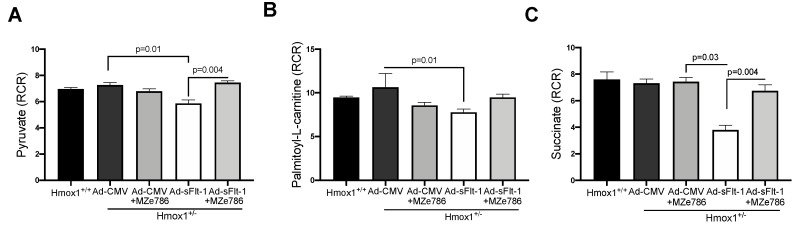
sFlt-1 inhibits cardiac mitochondrial activity in Hmox1 partial deficient mice. Mitochondrial oxygen consumption was measured in isolated cardiac mitochondria from Hmox1^+/−^ mice injected with Ad-sFlt-1 and Ad-CMV injected Hmox1^+/−^ mice exposed to MZe786 via gavage. The respiratory control ratio (RCR) (state 3/state 4o) was calculated using complex I-driven substrates: (**A**) pyruvate and (**B**) palmitoyl-L-carnitine. (**C**) RCR was calculated in complex II-stimulated cardiac mitochondria using succinate as substrate. Values are expressed as means ± SEM. *N* = 4.

To evaluate the capacity of Hmox1^+/−^ cardiac mitochondria for the oxidation of complex I substrates, we calculated the RCR in mitochondria exposed to pyruvate and palmitoyl-L-carnitine, respectively. The dose of Ad-sFlt-1 to administer was tested in-house. We observed that 0.5 × 10^9^ PFU/mL of Ad-sFlt-1 increased sFlt-1 circulating protein levels without inducing any toxic effect in mice, as shown in Figure S3. Ad-sFlt-1 injected mice showed a significant reduction in the calculated RCR in response to pyruvate, when compared to control animals injected with Ad-CMV (*p* = 0.0159). In Ad-sFlt-1 mice treated with MZe786, we evidenced the significant restoration of the pyruvate-dependent RCR in comparison to the Ad-sFlt-1 injected mice (*p* = 0.0043). No significant difference was evidenced between the Ad-CMV and Ad-sFlt-1 + MZe786 treated mice (*p* = 0.9), as shown in Figure 3A. In contrast, the calculated RCR resulting from palmitoyl-L-carnitine oxidation showed that Ad-sFlt-1 reduce the ability for the oxidation of fatty acids via complex I in comparison to control animals; however, this was not statistically different (*p* = 0.19). MZe786 enhanced the respiratory efficiency in cardiac mitochondria from Ad-sFlt-1 in comparison to Ad-sFlt-1 counterparts (*p* = 0.01). No difference was evidenced when comparing Ad-CMV with Ad-sFlt-1 + MZe786 treated mice (*p* = 0.9), as shown in Figure 3B. Regarding the cardiac mitochondrial complex II-dependent oxidation of succinate, our results showed that sFlt-1 significantly reduced the calculated RCR in comparison to control mice (Ad-CMV) (*p* = 0.03). MZe786 improved the respiratory efficiency in Ad-sFlt-1 injected Hmox1^+/−^ mice (*p* = 0.004) while no statistical difference was observed when compared to Ad-CMV (*p* = 0.9), as shown in Figure 3C. Furthermore, Ad-CMV, in the presence of MZe786, was similar to Ad-CMV alone, indicating that this drug causes no adverse effect, as shown in Figure 3. Oxygen consumption rates representative of state 3 and state 4o respiration were used to calculate the RCR, as shown in Figure S4. These observations suggest that environments of high sFlt-1 negatively regulate the cardiac mitochondrial activity in response to complex I substrate pyruvate and complex II substrate succinate in reduced HO-1 setting. Interestingly, the response to fatty acid substrate palmitoyl-L-carnitine was not impaired in cardiac mitochondria from Ad-sFlt-1 injected Hmox1^+/−^, suggesting that the fatty acid-driven mitochondrial function might play a key role in sustaining the energetic demands in Hmox1^+/−^ hearts. Therefore, events dysregulating this metabolic pathway might lead to mitochondrial dysfunction in environments of reduced HO-1. 

### 3.4. MZe786 Stimulates the Cardiac Mitochondrial Biogenesis and Antioxidant Defence in Hmox1^+/−^ Mice Exposed to High sFlt-1 Environment

**Figure 4:**
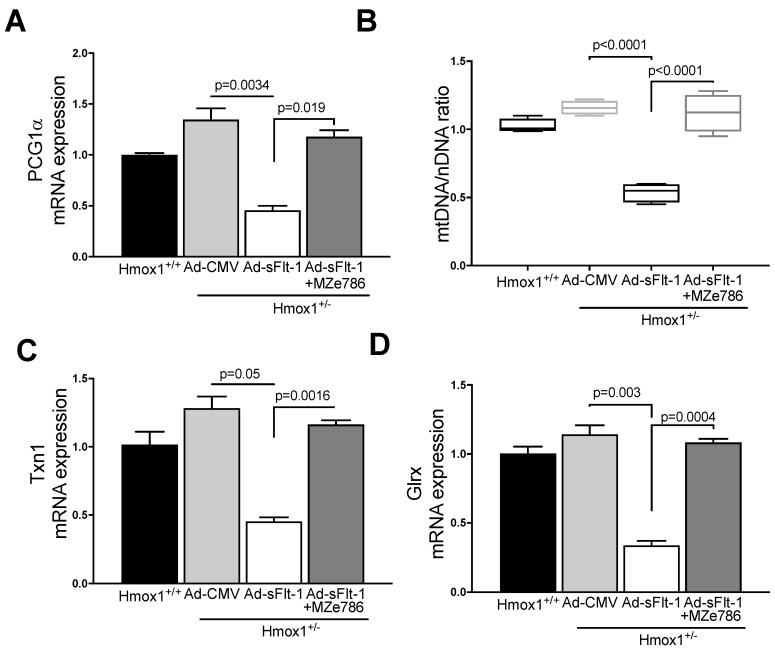
MZe786 stimulates the cardiac mitochondrial biogenesis and antioxidant defense in Hmox1^+/−^ mice in high sFlt-1 environment. (**A**) Relative PGC1α mRNA expression, (**B**) content of mitochondria expressed as the ratio of mitochondrial DNA by nuclear DNA (mtDNA/nDNA). (**C**) Relative mRNA expression of antioxidant gene thioredoxin (Txn1) and (**D**) glutaredoxin (Glrx) measured by qPCR in heart tissue from Ad-sFlt-1 and Ad-CMV injected Hmox1^+/−^ exposed to MZe786 via gavage. A, C and D values are expressed as means ± SEM. Values in B are expressed as median and whiskers represent maximum and minimum Values. *N* = 4.

Since MZe786 were shown to improve the mitochondrial biogenesis signal and antioxidant defense in Hmox1 knockout hearts, we explored whether MZe786 would exert similar effects in environments of high sFlt-1 and the partial loss of Hmox1. PGC1-α gene expression was significantly reduced in heart tissue from Ad-sFlt-1-injected Hmox1^+/−^ mice in comparison to Ad-CMV (*p* = 0.0034). Mice injected with Ad-sFl-1 and administered with MZe786 were able to significantly increase PGC1-α transcription in comparison to those that were given Ad-sFlt-1 alone (*p* = 0.019). The expression of cardiac PGC1-α was not different when Ad-CMV and Ad-sFlt-1 + MZe786 mice were compared (*p* = 0.24), as shown in Figure 4A. Consistently, Ad-sFlt-1 led to a reduced mtDNA/nDNA ratio in comparison to Ad-CMV (*p* < 0.0001), while MZe786 enhanced the cardiac mtDNA/nDNA in Ad-sFlt-1 + MZe786 treated mice (*p* < 0.0001). No statistical difference in the cardiac mitochondrial content was observed when Ad-CMV and Ad-sFlt-1 + MZe786 treated mice were compared (*p* = 0.43), as shown in Figure 4B.

Finally, we observed that cardiac tissue from Ad-sFlt-1-injected Hmox1^+/−^ mice showed the reduced transcription of antioxidant genes Txn1 and Glrx (*p* = 0.05 and *p* = 0.003, respectively) when compared to their wild type counterparts. The administration of MZe786 significantly stimulated Txn1 and Glrx gene expression in the hearts from Ad-sFlt-1-injected Hmox1^+/−^ mice (*p* = 0.0016 and *p* = 0.0004, respectively). No statistical difference between Txn1 and Glrx gene expression was observed when compared to Ad-CMV (*p* = 0.55 and *p* = 0.21, respectively), as shown in Figure 4C,D. These observations suggest that MZe786 enhances the transcription of cardiac antioxidant genes in response to high sFlt-1 and low HO-1 milieu.

## 4. Discussion

Several studies have demonstrated that the risk of developing cardiovascular disease is significantly higher in women with a history of preeclampsia [57,58,59]. In particular, Wu et al. showed that women who experienced preeclampsia have a 4.2-fold increased risk of heart failure, 2.5-fold increased risk of coronary artery disease and 1.8-fold increased risk of stroke [60]. In addition, the severity of preeclampsia has been linked to a greater long-term risk of developing cardiovascular disorders [61]. Here, we explored the effects of the hydrogen sulfide-releasing molecule, MZe786, in protecting the cardiovascular mitochondrial function and antioxidant machinery in a low HO-1 and high sFlt-1 setting, mimicking the molecular impairments of a preeclampsia-like condition in vivo. Our results provide novel insights into the role of HO-1 in modulating mitochondrial OXPHOS and antioxidant gene expression and suggest that MZe786 protects the cardiovascular antioxidant capacity. MZe786 may have therapeutic potential for preeclampsia-induced long-term cardiovascular disease.

Two decades ago, we demonstrated that HO-1 protein expression is reduced in placentas from pregnancies complicated with preeclampsia [62]. More recent studies have shown that the deletion of the Hmox1 gene in mice impairs placentation. Likewise, the partial deletion of Hmox1 also leads to signs of intrauterine growth restriction [63]. In humans, the variation in maternal and fetal microsatellites in the Hmox1 promoter is likely to predispose women to preeclampsia during pregnancy [64]. In the present study, Hmox1 deficiency resulted in the suppression of the mitochondrial activity and reduced PGC1-α gene expression in the heart. In 2005, Lui et al. reported that Hmox1 deficient mice displayed increased signs of myocardial injury after ischemia/reperfusion insults [27]. Interestingly, in a murine model of sepsis, the controlled delivery of CO resulted in improved mitochondrial energetics and an enhanced mitochondrial biogenesis signal in the heart [65]. These suggest that HO-1, possibly through its active byproducts, is able to sustain the energetics of cardiac mitochondria, and the dysregulation of the HO-1 pathways may impair the expression of PGC-1α, resulting in reduced mitochondrial content.

The myocardial tissue has the ability to metabolize different substrates such as fatty acids, amino acids, glucose, lactate, and ketone bodies, depends on the bioavailability of substrates in the cardiac environment [66]. Typically, 60–90% of mitochondrial ATP production is generated by fatty acid metabolism, whereas 10–40% comes from pyruvate oxidation [67]. Our observations showed that the loss of HO-1 results in mitochondrial dysfunction in the heart. Our study focused on evaluating the rate of mitochondrial utilization in response to mitochondrial substrates. This approach allowed us to evidence the extent to which HO-1 deficiency and sFlt-1 would impair the ability of mitochondria to perform its metabolic activities.

In response to complex I substrates, pyruvate and palmitoyl-L-carnitine, we evidenced a reduced respiratory capacity in Hmox1^−/−^ mice. In the case of pyruvate, it has been shown that its metabolism requires mitochondrial import mediated by the carrier-regulated process [68]. Likewise, fatty acid oxidation depends on the carnitine shuttle transport system to the inner mitochondrial matrix coupled to β-oxidation [69]. Interestingly, mitochondrial complex II has distinguished characteristics linking the electron transport chain and tricarboxylic acid cycle in mammalian cells. It has been reported that the activation of complex II enhances the reserve respiratory capacity in myocytes [70]. Our results showed reduced RCR in Hmox1-deficient hearts in response to succinate, suggesting that the loss of HO-1 would impair the maximal respiratory capacity in the cardiac tissue. Together, these results suggest that HO-1 might regulate mitochondrial substrate uptake and the activation of its complexes by modulating the mitochondrial genesis signal through PGC1-α.

The delivery of sFlt-1 to Hmox1^+/−^ mice further evidenced the role of sFlt-1 to impair the cardiac mitochondrial activity in a reduced HO-1 setting. In these experiments, non-pregnant females were exposed to high sFlt-1. As Akhter and colleagues previously reported, circulating levels of sFlt-1 remain higher in women post preeclampsia even one year after delivery (25). The administration of sFlt-1 to non-pregnant animals results in high blood pressure, proteinuria, and endotheliosis, characteristics of preeclampsia [71]. Therefore, our approach emulated the effects of acute sFlt-1 exposure to HO-1 defective environment, mimicking the molecular milieu in a preeclampsia-like condition.

The efficiency of the cardiac mitochondrial activity was significantly impaired in sFlt-1-treated Hmox1^+/−^ mice. This was accompanied by reduced PGC1-α gene transcription and mitochondrial content in comparison to the control. These observed effects were consequently attributable to sFlt-1-induced damage to the mitochondria. We recently demonstrated that sFlt-1, dose-dependently disturbs the endothelial cellular bioenergetics and promotes mitochondrial specific reactive oxygen species production [45]. Likewise, others have reported that in mice, overexpression of sFlt-1 leads to mitochondrial swelling and oxidative stress in the placenta [72]. Based on this evidence, our results indicate that sFlt-1 is damaging to the mitochondria of cardiac tissue and imply that sFlt-1-induced cardiac mitochondrial dysfunction is a potential molecular mechanism of cardiovascular disease in women, post preeclampsia.

Hydrogen sulfide donors have been demonstrated to exert protective effects on the cardiac oxidative metabolism in different scenarios of cardiovascular disease [73,74,75,76] and may involve an interplay and crosstalk between nitric oxide, H_2_S, and carbon monoxide, the gaseous product of HO-1. H_2_S may have a role in increasing the expression of HO-1 and, subsequently, the CO level, therefore excreting protective effect. A proposed mechanism for this involves the Keap1/Nrf2 pathway. The persulfidation of Keap1 Cys_151_ by the reaction of H_2_S with oxidized Cys_151_ by H_2_S-derived HS· leads to the dissociation and nuclear translocation of Nrf2, ultimately resulting in an increased HO-1 protein level [39,77,78]. Endothelial dysfunction is improved by H_2_S, which is produced in endothelial cells and participates in the fine regulation of endothelial integrity and functions [79,80]. Disturbed H_2_S bioavailability was suggested to be a novel indicator of endothelial dysfunction progression and is manifested in different forms in multiple pathologies, including preeclampsia [80]. Therapeutics, aimed at remedying altered H_2_S bioavailability, may benefit diseases with unmet medical needs.

This study shows that the hydrogen sulfide releasing molecule, MZe786, protects cardiac mitochondrial efficiency through complexes I- and II-driven OXPHOS and improves antioxidant gene transcription in low HO-1 settings. As previously shown, exogenous hydrogen sulfide donors sustain the mitochondrial membrane potential, inhibit apoptosis, and suppress reactive oxygen species generation in a model of cardiac hypertrophy in mice [81]. Furthermore, the metabolism of hydrogen sulfide is linked to the mitochondria, while its targeted delivery to the mitochondria reduces infarct size and suppresses mitochondrial damage in mice [82]. Likewise, the activation of hydrogen sulfide pathways has been shown to protect against sFlt-1-induced renal damage [83]. These results suggest that hydrogen sulfide donors may exert protective effects on the cardiac respiratory capacity in HO-1 compromised models, possibly by modulating the cardiac mitochondrial content via PGC1-α, impaired in HO-1 deficient settings and exacerbated in a high sFlt-1 environment.

Interestingly, hydrogen sulfide-induced cardio-protection has been linked to enhanced antioxidant defenses via the upregulation of reduced glutathione levels [84] and Trx-1 [85]. Particularly, Nicholson et al. reported that hydrogen sulfide increased the cardiac levels of the Txr-1 gene in a murine model of ischemia-reperfusion injury [85]. Similar to these reports, our study demonstrates that the hydrogen sulfide-releasing molecule, MZe786, improves the antioxidant capacity of the heart via the upregulation of Trx-1 gene transcription. Similarly, another important regulator of intracellular redox signaling is Glrx. Glrx maintains protein thiols in a reduced state [86] and regulates apoptosis in cardiomyocites [87]. Glrx is expressed in the mitochondria [88]. Hence, the protective effects of MZe786 on mitochondrial activity might be associated with increased Glrx transcription. Together, our observations denote that MZe786, potentially though its hydrogen sulfide-releasing moiety, enhances antioxidant defenses by upregulating the transcription of redox signaling regulators, Trx-1 and Glrx, in cardiac tissue impaired by the partial loss of HO-1 and high sFlt-1.

Our study shows that MZe786 improved mitochondrial capacity when the high sFlt-1 environment was accompanied by the partial or complete loss of HO-1 function in animal models. Although the total deficiency of Hmox1 resulted in a marked reduction in mitochondrial oxidative capacity, MZe786 improved the respiratory efficiency and restored antioxidant defenses to levels similar to the Hmox1^+/+^ mice. Similarly, the partial loss of Hmox1 reduced the mitochondrial capacity in comparison to the total loss of HO-1, even though the reduction in the respiratory efficiency was less pronounced. These observations suggest that HO-1 is necessary to support the cardiac mitochondrial function. Although, one allele provides protection, as demonstrated by the cardiac mitochondrial function, in high sFlt-1, this protection is abrogated. MZe786 also improved the respiratory capacity in these low HO-1 and high sFlt-1 environments, allowing for the protection of the mitochondrial activity in response to mitochondrial substrates, pyruvate, palmitoyl-L-carnitine, and succinate. Together, these observations demonstrate that MZe786 can provide beneficial effects on the cardiac metabolism, allowing for the restoration of the mitochondrial activity and providing antioxidant-enhanced protection.

Here, we provide first-time evidence demonstrating that the hydrogen sulfide-releasing molecule, MZe786, enhances cardiac mitochondrial activity and protects the antioxidant capacity in a reduced HO-1 and high sFlt-1 environment, mimicking the molecular impairments encountered in preeclampsia. Our results provide new insights into the molecular mechanisms associated with long-term cardiovascular disease observed in in women post preeclampsia and suggest MZe786 as an effective therapeutic molecule to relieve the sFlt-1-induced mitochondrial damage in the heart.

## 5. Conclusions

Deficiency in HO-1 disturbed the mitochondrial activity and these effects were associated with reduced mitochondrial content and suppressed the antioxidant capacity. This suggests that the loss of HO-1 may impair the cardiac tissue ability to sustain metabolic demands through the mitochondria and to respond to oxidative stress-induced damage. In these settings, MZe786 was shown to protect the antioxidant capacity and mitochondrial function, suggesting that this molecule may be beneficial in protecting cardiac tissue against events leading to impairments in the antioxidant capacity of the heart. Consequently, the partial deficiency of HO-1 in a high sFlt-1 environment provided a more realistic setting, mimicking the preeclampsia-like molecular defects in the heart. Soluble Flt-1 impaired the mitochondrial activity and suppressed antioxidant gene transcription in the cardiac tissue. The administration of MZe786 protected the mitochondrial activity and upregulated the antioxidant genes. Our results suggest that MZe786 protects the heart from sFlt-1-induced mitochondrial damage in an environment of low HO-1 via the upregulation of key antioxidant genes and by exerting protective effects on the mitochondrial activity in the hearts.

## Supplementary Materials

The following are available online at https://www.mdpi.com/2076-3921/9/7/598/s1, Figure S1: Chemical structure of H2S releasing aspirin, MZe786 [2-acetyloxybenzoic acid 4-(3-thioxo-3H-1,2-dithiol-5-yl)phenyl ester]. Figure S2: Respiration parameters from Hmox1 deficient mice. Figure S3: Plasma level of sFlt-1 six days post-injection. Figure S4: Respiration parameters from Hmox1^+/−^ mice.
