# Supplementary material for: MZe786 Rescues Cardiac Mitochondrial Activity in High sFlt-1 and Low HO-1 Environment
Source: Antioxidants (Basel). 2020 Jul 9;9(7):598. doi: 10.3390/antiox9070598 (PMC7402164; doi:10.3390/antiox9070598)
Supplement: Supplementary file 1 [file antioxidants-09-00598-s001.pdf]

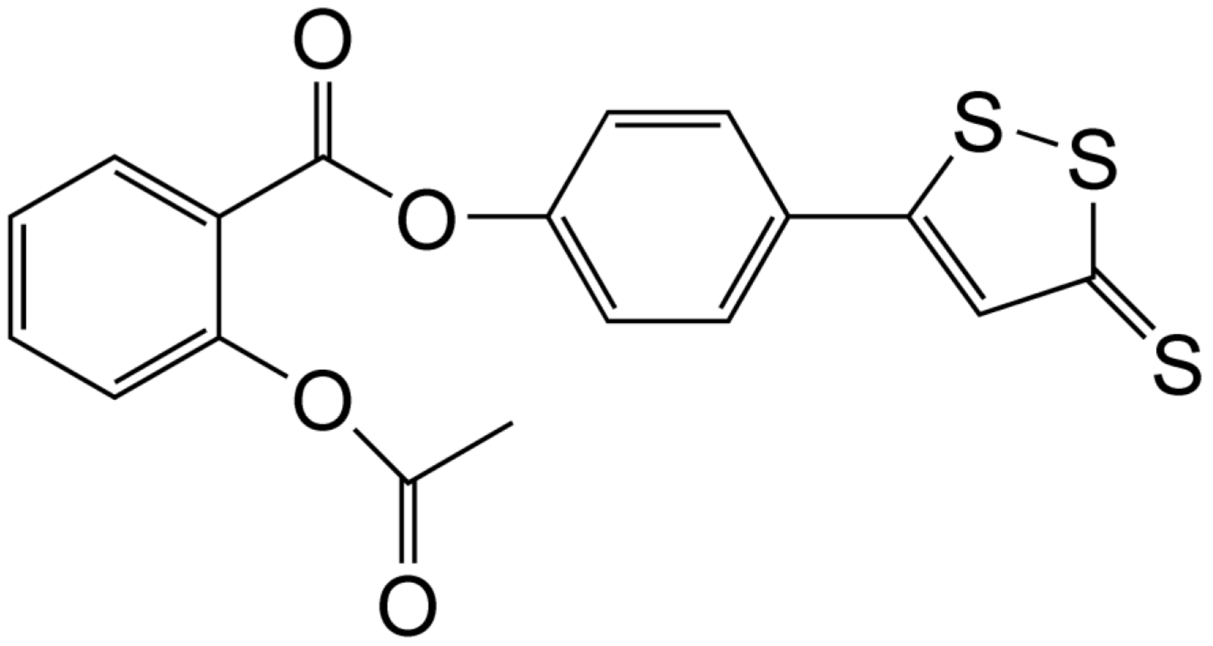

Supplementary Figure 1. Chemical structure of H2S releasing aspirin, MZe786 [2-acetyloxybenzoic acid 4-(3-thioxo-3H-1,2-dithiol-5-yl)phenyl ester].

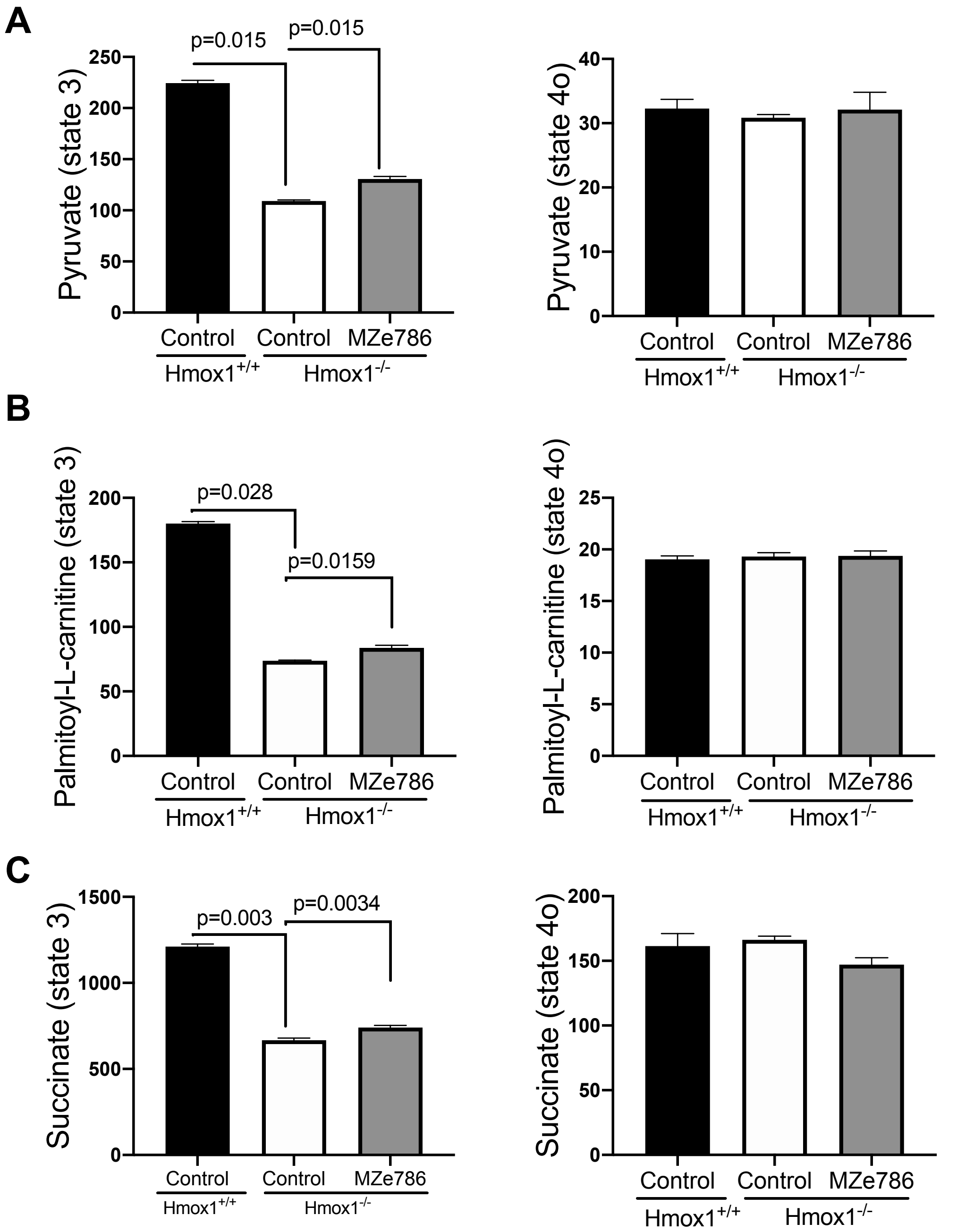

Supplementary Figure 2. Respiration parameters from Hmox1 deficient mice. Oxygen consumption rates representative of ADP-stimulated (state 3) and non-ADP-stimulated (state 4o) respiration in cardiac isolated mitochondria in response to substrates **(A)** pyruvate, **(B)** palmitoyl-L-carnitine and **(C)** succinate. n=4.

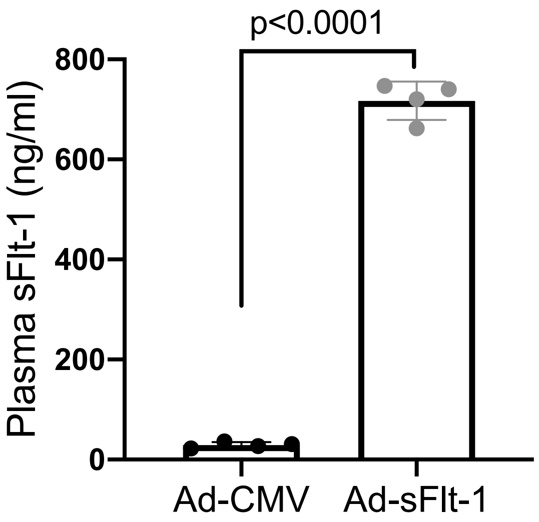

Supplementary Figure 3 . Plasma level of sFlt-1 six days post-injection. Hmox1$^{+/-}$ mice were injected with either Ad-CMV or Ad-sFlt-1 via tail-vein injection and the plasma level of sFlt-1 were measured by ELISA. Values are expressed as means ± SEM. Values in B are expressed as median and whiskers represent maximum and minimum values. n=4.

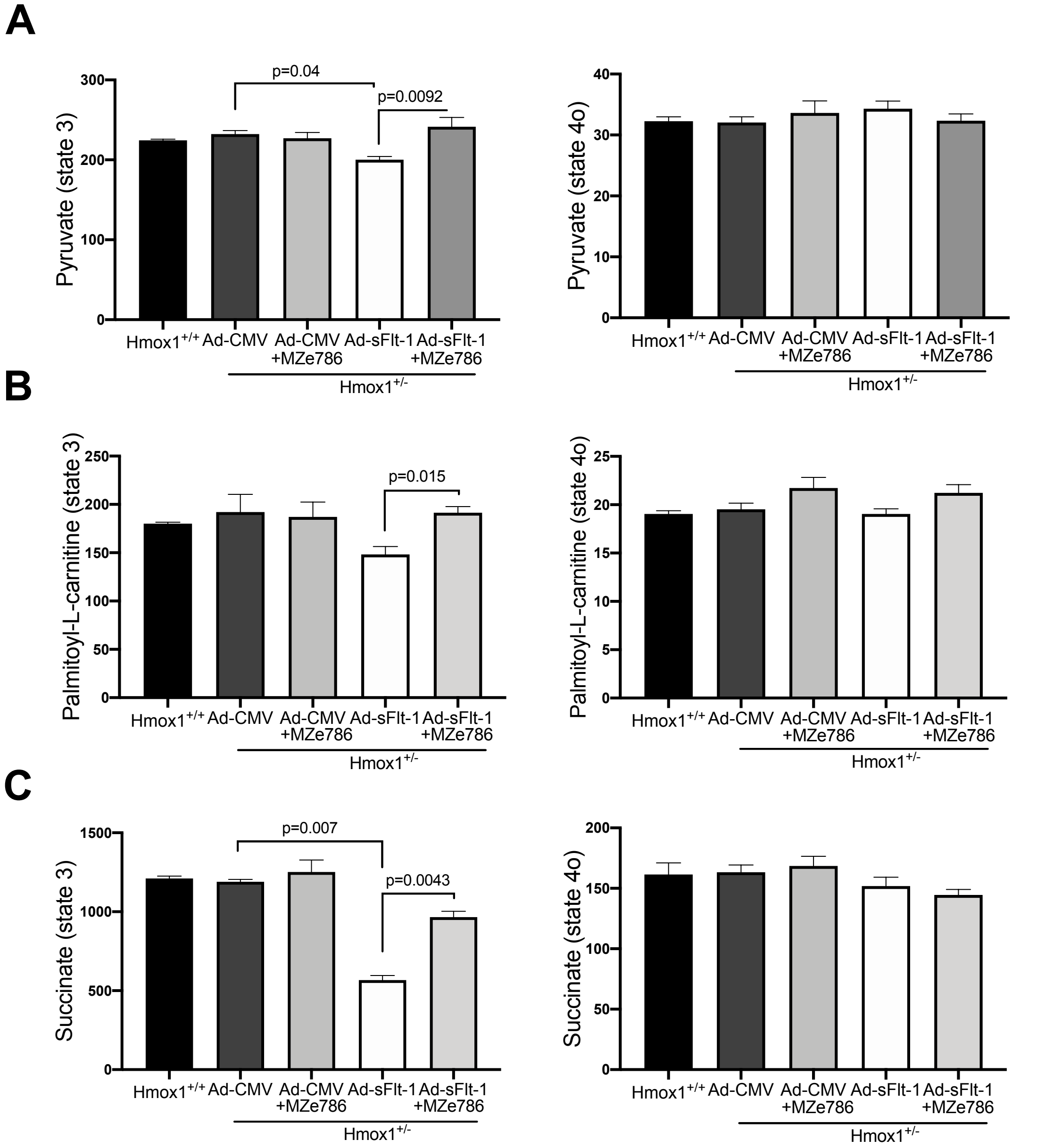

Supplementary Figure 3. Respiration parameters from Hmox1$^{+/-}$ mice. Oxygen consumption rates representative of ADP-stimulated (state 3) and non-ADP-stimulated (state 4o) respiration in cardiac isolated mitochondria in response to substrates **(A)** pyruvate, **(B)** palmitoyl-L-carnitine and **(C)** succinate. n=4.
